# Navigating the EU AI Act: implications for regulated digital medical products

**DOI:** 10.1038/s41746-024-01232-3

**Published:** 2024-09-06

**Authors:** Mateo Aboy, Timo Minssen, Effy Vayena

**Affiliations:** 1 Centre for Law, Medicine, and Life Sciences (LML), Faculty of Law, University of Cambridge, Cambridge, UK; 2 Translational Health Sciences, Medical Sciences Division, University of Oxford, Oxford, UK; 3 Centre for Advanced Studies in Bioscience Innovation Law, Faculty of Law, University of Copenhagen, Copenhagen, Denmark; 4 Department of Health Sciences and Technology, ETH Zurich, Zurich, Switzerland

**Keywords:** Law, Policy, Business, Technology

## Abstract

The newly adopted EU AI Act represents a pivotal milestone that heralds a new era of AI regulation across industries. With its broad territorial scope and applicability, this comprehensive legislation establishes stringent requirements for AI systems. In this article, we analyze the AI Act’s impact on digital medical products, such as medical devices: How does the AI Act apply to AI/ML-enabled medical devices? How are they classified? What are the compliance requirements? And, what are the obligations of ‘providers’ of these AI systems? After addressing these foundational questions, we discuss the AI Act’s broader implications for the future of regulated digital medical products.

## Introduction

The integration of artificial intelligence (AI) into regulated digital medical products is expected to transform the landscape of healthcare diagnosis. Both US and European Patent Office data show robust and rising patenting of medical AI inventions, reflecting an increased level of innovation at the invention stage^1^. These developments are not limited to early-stage innovation; they are now paralleled by a significant surge in actual practical applications of AI in healthcare, resulting in the development and market introduction of a new generation of commercially available medical AI products. The number of medical devices incorporating AI/ML continues to increase. In fact, the U.S. Food and Drug Administration (FDA) has already reviewed and authorized over 690 AI/ML-enabled medical devices, underscoring the growing importance of this technology in clinical settings^2^.

Legal regulation of AI will play a big part in the future of regulated digital medical products, including medical devices^3^. The overarching legal framework surrounding AI/ML-enabled medical products is crucial, as it shapes their development, authorization, market introduction, deployment, and use. Until recently, medical devices were regulated primarily by sector-specific laws and regulations such as the FDA medical device law and the European Union (EU) Medical Devices Regulation (MDR)^4^. This has now changed. The EU has just approved the world’s first comprehensive legal framework for AI, the EU AI Act^5^. In this context, the EU AI Act emerges as a pioneering legislative framework, marking the first comprehensive and general legal regulation of AI globally. Similar to the impact of the EU General Data Protection Regulation (GDPR) on any product or service involving personal data, the EU AI Act is poised to have a profound impact on the future of AI across industries. In this paper, we analyze the EU AI Act’s impact on regulated digital medical products.

## What is the EU AI Act?

The EU AI Act is a landmark regulation, representing a significant step towards the legal governance of AI technologies. The Act was approved on 13 March 2024. Despite being branded as an “Act,” it is, in fact, an EU Regulation (Regulation EU 2024/1689). As such, the EU AI Act shares the same legal instrument and standing as the EU Medical Device Regulation (MDR)^4^, EU In Vitro Diagnostic Medical Devices Regulation (IVDR)^6^, EU Clinical Trials Regulation (CTR)^7^, and the EU General Data Protection Regulation (GDPR)^8^, four European regulations that have influenced regulated digital medical products. The general-purpose AI obligations will apply 12 months after entry into force in May 2025. The obligations on high-risk AI systems will apply 24 to 36 months after entry into force, depending on the specific type of high-risk AI. For regulated digital medical products subject to the EU MDR/IVDR (Annex II), the obligations for their AI systems will apply 36 months after entry into force. In general, the obligations for high-risk systems intended to be used as a safety component of a product (or the AI is itself the product) that is required to undergo a third-party conformity assessment under existing EU laws will apply 36 months after entry into force.

In the EU legislative context, an EU Regulation is a binding legislative act applicable in all EU countries. Unlike Directives, which generally require individual countries to enact their own laws within a given timeframe to achieve certain objectives, Regulations are directly enforceable as law across the EU. This means that the EU AI Act, as a Regulation, will have immediate legal effect in all EU member states without the need for separate national legislation. Initially proposed by the European Commission in 2021, the EU Act went through significant developments, culminating in a political agreement between the Council and the European Parliament in December 2023 and the Council of EU Ministers in February 2024. This agreement marked a major milestone in the EU’s ambition to become the first region globally to adopt comprehensive legislation on AI.

The EU AI Act’s recitals lay out its foundational principles and objectives. These include: (1) ensuring AI systems’ safety and respect for fundamental rights, (2) promoting AI innovation and uptake within the EU; (3) creating legal certainty to facilitate investment and innovation in AI; and (4) addressing risks associated with specific uses of AI, particularly those posing high risks to fundamental rights.

As opposed to sector-specific law that regulate a specific type of product, such as the EU MDR^4^ that imposes requirements for market access, safety, and effectiveness of medical devices^9^, the AI Act has a broader scope. Similarly to the GDPR, the AI Act’s structure is comprehensive and general (Box 1). The Act complements the GDPR by addressing the unique risks posed by AI technologies, including those related to data processing^10^.

Box 1 Overview of EU AI Act’s Structure Relevant to Regulated Digital Medical Products such as Medical Devices **Classification of AI Systems:** AI systems are categorized based on the level of risk they pose, from unacceptable and high-risk to limited and minimal risk. For digital medical products, the focus is primarily on high-risk AI systems, which include those used in medical devices subject to EU MDR/IVDR.

**Risk-Based Classification:** The AI Act introduces a risk-based approach to AI regulation, classifying AI systems into four categories:

- Prohibited AI (e.g., social credit scoring)
- High-risk AI systems (e.g., medical devices)
- General-purpose AI and foundation models (e.g., generative AI, LLMs)
- Low-risk AI systems

Prohibited AI includes practices such as social scoring and manipulative AI, which the legislation seeks to ban outright.

**Requirements for High-Risk AI Systems:** These include strict compliance obligations such as risk management, data governance, transparency, and human oversight. Digital medical products falling under high-risk AI systems must adhere to these requirements.

**Applicability and Territorial Scope**: The AI Act applies to providers placing AI systems in the EU market, users within the EU, and also outside providers/users if their AI system outputs are used in the EU. This extraterritorial reach is similar to the GDPR.

**Interaction with Other Regulations:** The Act interacts with existing regulations like the EU MDR, IVDR and GDPR. For digital medical products, this means navigating a complex regulatory landscape where AI-specific requirements (from the AI Act) intersect with medical device (EU MDR/IVDR) and data protection regulations (GDPR).

**Incident Reporting and Post-Market Monitoring:** Developers of high-risk AI systems, including those used in medical products, are required to set up reporting systems for serious incidents and engage in continuous post-market monitoring.

## How does the EU AI act apply to regulated digital medical products?

The EU AI Act is a pivotal regulation for digital medical products, impacting all AI/ML-enabled devices and systems. These include medical devices, diagnostics, and regulated clinical support tools, ranging from stand-alone software as medical devices (SaMD) and AI as medical devices (AIaMD) to complex hardware-software combinations. Generally, these digital medical products are regulated by the EU MDR^4^ and the EU IVDR^6^. As such, these devices are already subject to stringent sector-specific regulations requiring robust compliance standards^9^. Additionally, regulated digital medical products often process personal health data and therefore need to also comply with the GDPR. The EU AI Act’s extra-territorial reach is akin to the GDPR^11^, transcending European borders and impacting international AI system providers and deployers. It applies to ‘providers placing on the market or putting into service AI systems or placing on the market general-purpose AI models in the Union, irrespective of whether those providers are established or who are located within the Union or in a third country’ and providers and deployers established outside the EU if ‘the output produced by the system is used in the EU’ (Art. 2). Thus, we expect the majority of the international manufacturers of the 690 AI/ML-enabled medical devices authorized by the US FDA will be within its scope and will be materially impacted from a regulatory compliance standpoint.

### How are regulated digital medical products classified?

Under the EU AI Act, AI systems in regulated digital medical products, such as those in AI/ML-enabled medical devices, are classified as “high-risk” (Art. 6, Annex II). This is the highest risk classification for permitted uses of AI. And it triggers a cascade of compliance requirements (Art. 8). Risk management becomes a focal point (Art. 9), intertwining with the EU MDR risk-management system to identify, evaluate, and mitigate the ‘reasonably foreseeable risks’ that high-risk AI systems can pose to health, safety, or fundamental rights such as privacy and data protection. Data governance (Art. 10), a critical aspect, aligns with GDPR to safeguard patient data, but it is materially extended with AI-specific requirements related to training, validation, and testing data sets. This includes ‘an assessment of the availability, quantity and suitability of the data sets that are needed’, ‘examination in view of possible biases that are likely to affect the health and safety of persons’, and ‘take into account […] the characteristics or elements that are particular to the specific geographical, contextual, behavioural or functional setting within which the high-risk AI system is intended to be used’ (Art. 10). Providers established outside the EU will need to appoint an EU authorized representative (Art. 25).

### What are the obligations of providers of medical AI systems?

The EU AI Act delineates specific obligations for providers of high-risk AI systems. As expected, providers are responsible for ensuring their high-risk systems are compliant with the above requirements (Art. 16). The Act specifies that providers must implement an AI quality management system (AI QMS) to ensure compliance (Art. 17). The required AI QMS is the foundational building block to ensure ongoing quality and compliance (Art. 17). This is because the AI QMS brings together the required AI documentation (Art. 18), conformity assessments and automatically generated logs (Art. 20), corrective actions and duty of information (Art. 21), post-market monitoring (Art. 61), reporting serious incidents (Art. 62), internal and external audits, and cooperation with the competent authorities (Art 23) to achieve regulatory compliance.

### What are the technical documentation requirements? Is it possible to combine them within the medical technical file?

Similar to the EU MDR, the EU Act requires a “technical documentation” file for the AI system to ‘demonstrate the high-risk AI systems complies with the requirements’ (Art. 11). This is needed even if the AI system is just a minor sub-component of the digital regulated product. Since it is used as part of a medical device or IVD, it is still considered high-risk according to the Art 6 classification rules. The technical documentation of a high-risk AI system needs to be completed and available before the system is ‘placed on the market or put into service’ and must be kept updated (Art. 11).

The technical documentation for high-risk AI systems is comprehensive. It is substantially more than the documentation needed for US FDA authorization of AI/ML-enabled medical devices, especially those authorized through the 510(k) or De Novo pathways for moderate-risk devices^3,12^. It includes detailed descriptions of the elements of the AI system and the process of its development including design specification; system architecture; key design choices as well as their rationale and assumptions made; data requirements; training methodologies; computational resources used to develop, train, and validate the AI system; validation and testing procedures; and performance metrics (Annex IV). Thus, regulated digital medical products that include an AI system need to provide the required Annex IV technical documentation for the AI system in addition to the EU MDR/IVD technical documentation. That said, Art.11(2) provides the legal basis to create and maintain a single technical documentation file containing the combined information. Accordingly, we expect that medical device manufacturers will rely on Art. 11(2) to leverage their EU MDR/IVDR technical documentation system for their regulated products and simply expand the documentation of the AI system or sub-system by appending the Annex IV information. A concern is that the high-bar documentation requirements for the AI system could be detrimental to new entrants. The EU AI Act attempts to address this concern by enabling SMEs, including start-ups, to provide simplified technical documentation -still to be developed by the Commission- ‘targeted at the needs of small and micro enterprises.’

Traceability through record-keeping and automatic logging capabilities over the lifetime of the system (Art. 12), AI-system transparency (Art. 13), and human oversight (Art. 14) are emphasized with prescribed requirements aimed at ensuring safe and ethical AI deployment. Finally, Art. 15 specifies design and development requirements for high-risk AI systems regarding accuracy, robustness, cybersecurity, and resilience throughout their lifecycle. As with Art 13 (transparency) emphasis is placed on the provision of information such as full disclosure of the levels of accuracy, including its metrics, which the ‘AI system has been tested and validated and which can be expected’, as well as ‘any known foreseeable circumstances that may have an impact on the expected level of accuracy, robustness and cybersecurity.’

### What are the requirements for the AI Quality Management System? And can it be integrated with the medical device QMS?

Manufacturers of regulated digital products such as medical devices already have existing obligations to implement a QMS to satisfy the requirements of EU MRD/IVDR. Given that these manufacturers will already have implemented the international consensus standard for medical devices (ISO/IEC 13485 Medical Device QMS), would they have to implement another parallel AI QMS to manage their AI-systems (e.g., an AI sub-system within their medical device or AI SaMD)? For instance, would they be expected to also implement the ISO/IEC 42001 AI QMS? Not necessarily. The EU Act states: ‘for providers of high-risk AI systems that are subject to obligations regarding quality management systems […] under sector Union law, the aspects described […] may be part of the quality management systems pursuant to that law’ (Art. 17). Thus, it is expected that medical device manufacturers will continue using the ISO/IEC 13485 QMS and incorporate the requirements of Art. 17 within their existing medical device QMS functions.

Providers (Art. 16), authorized representatives (Art. 25), manufacturers, importers (Art. 26), distributors (Art. 27), and deployers (Art. 29) face distinct responsibilities. The overarching goal is to ensure a high standard of compliance throughout the product lifecycle and along the entire AI value chain (Art. 28). The Act also establishes a comprehensive framework for standards of conformity, including an EU declaration of conformity (Art. 48), CE marking of conformity (Art. 49), registration (Art. 51), and an EU Database for certain high-risk AI systems, enhancing oversight and transparency^13^.

Table 1 provides a summary of the key EU AI articles applying to AI/ML-enabled regulated digital medical products. The Act presents both challenges and opportunities for medical AI/ML providers. Compliance with the requirements will likely necessitate a revamp of existing practices, particularly on technical documentation of the AI design, validation, and performance testing within an overall AI-specific risk management framework embedded in a medical device QMS. That said, the peer-reviewed literature and open-source literature (medical AI papers in arXiv, open-source models, and open-source data) shows that many AI developers are already implementing these AI development best practices from the technical standpoint. Thus, these AI system developers are well suited to comply with the AI EU Act’s obligations as providers of high-risk AI systems. Such compliance can be a source of significant competitive advantage, especially if it helps increase acceptability, adoption and trust among deployers, healthcare professionals, and consumers. Yet, recent scholarship has critically examined the conceptualization of trust in the AI Act and criticized its simplistic conflation of trustworthiness (as in “Trustworthy AI”) and the acceptability of risks^14^.

**Table 1:** Summary of EU AI Act’s key articles applying to AI/ML-enabled regulated digital medical products

Aspect	Application to Medical Devices	Relevant EU AI Act Article(s)
Classification of AI Systems	Medical devices with AI are classified as high-risk and must adhere to strict regulatory requirements, ensuring their safety and effectiveness.	Art. 6
Risk Management System	Medical device manufacturers must implement comprehensive AI-specific risk management systems, aligning with EU MDR/IVDR standards.	Art. 9
Data Governance	Manufacturers must ensure data governance in compliance with GDPR, focusing on data protection and privacy in healthcare AI.	Art. 10
Technical Documentation	Detailed technical documentation required for the AI-system, similar to EU MDR for the medical device, to demonstrate compliance and safety of AI medical devices.	Art. 11
Transparency & Human Oversight	Provides must ensure transparency in AI operations and maintain human oversight, adhering to ethical standards.	Arts. 13, 14, 52
Obligations on Providers	Providers, including manufacturers, must implement a QMS and maintain detailed AI documentation for regulatory compliance. The AI QMS and be integrated with the Medical Device QMS (e.g., ISO13485)	Arts. 16, 17, 18
General Purpose AI Models	Providers and deployers of general-purpose AI models (GPAI) including generative AI systems (e.g., LLMs) have specific transparency obligations, inducing providing GPAI-specific technical documentation.	Art. 52–55
Conformity Assessment, Logs, CAPA	Manufacturers must conduct conformity assessments, maintain logs, and implement CAPA for robust and compliant AI medical devices.	Arts. 19–23
Notified Bodies	Manufacturers must engage with notified bodies for certification and regular monitoring of high-risk AI systems.	Arts. 30–38
Obligations on Product Manufacturers	Manufacturers are responsible for ensuring the compliance, efficacy, and safety of their AI medical devices.	Art. 25, 43
Obligations of Deployers, Importers and Distributors	Importers and distributors must ensure that AI medical devices meet EU standards and regulations before entering the market.	Arts. 24–27
Standards of Conformity	Compliance with established standards of conformity; EU Declaration of Conformity required for market access.	Arts. 40–44, 47, 48–49
Post-Market Monitoring	Ongoing monitoring of AI medical devices post-market, ensuring continued compliance and addressing safety concerns.	Arts. 61, 62
Enforcement	Compliance with the Act’s enforcement mechanisms to manage non-compliance and safeguard public health and safety.	Arts. 63, 70–72

## Discussion

The EU AI Act’s overarching goals emphasize enhancing safety, transparency, and accountability for AI systems in regulated digital products. The Act’s comprehensive approach seeks to set a global benchmark for AI regulation, emphasizing ethical considerations and the responsible deployment of AI technologies. It also aims to establish a regulatory environment that promotes these objectives while encouraging innovation. But, will it achieve this dual objective? What are the foreseeable risks of the EU AI Act? Navigating the line between fostering innovation that promotes technological advancement while ensuring that such innovations do not compromise other regulatory goals is challenging. Yet, getting this balance right is crucial for Europe’s competitiveness in the digital healthcare market^15^.

Ideally, the EU AI Act will encourage the development of advanced AI/ML medical devices and other regulated AI digital products that comply with stringent safety and effectiveness standards. However, there are also potential downsides.

First, developers and providers of AI/ML medical devices will have to conform with regulatory requirements of both the EU MDR and the AI Act. While there is some overlap with EU MDR requirements that can be useful for EU AI Act compliance (e.g., risk assessment^16^, QMS, technical file, post-marketing surveillance), what similar requirements mean under the different regulations might cause confusion. Specificity and alignment in requirements that may appear similar and overlapping will likely be more burdensome for SMEs which often have to prioritize their limited resources towards engineering, quality, and product development, as opposed to maintaining a large regulatory team capable of navigating and handling the additional regulatory complexity. A recent study showed that medical device companies are already having significant difficulties implementing the EU MDR; the key challenges cited include additional workload for technical documentation, higher resource expenditure and cost increase, lack of clarity regarding regulatory requirements, and delays caused by a lack of availability of notified bodies. The findings reveal that MDR is seen as a challenge for all businesses regardless of size, but especially for SMEs, which are often ‘overwhelmed by the necessary additional expenditure’ and ‘the increased requirements resulting from the MDR are so extensive that it is considered to be an existential threat’ resulting in a reduction of the product portfolio, inability to bring new products to market in the EU, or withdrawal of medical devices from the EU market^17^. Given that the EU AI Act adds significant regulatory requirements on top of the high compliance requirements already placed by the EU MDR, new medical AI start-ups and small enterprises with limited resources might be disproportionally affected, despite provisions in the AI Act to support SMEs. Currently, European-headquartered corporations are among the top medical AI/ML patent owners, indicating a degree of European innovation leadership at the invention stage^1^. Could the EU AI Act undermine this leadership position? Future research should empirically evaluate these trends and compare this measure of innovation activity before and after the EU AI Act is operational^18^.

Second, the successful implementation of the EU AI Act requires concerted efforts from a broad spectrum of stakeholders, including policymakers, regulators, notified bodies, AI providers and AI deployers, industry, and the public sector. This presents potential risks, including the risk of bottlenecks and lack of synchronicity. Even if AI providers and manufacturers of regulated digital medical products are ready to comply with its stringent requirements, there may be challenges due to lack of capacity or readiness level from other stakeholders such as the availability of notified bodies. As an example, it has been several years since the introduction of the EU MDR/IVDR. Yet, the lack of capacity of notified bodies to certify medical devices has hindered their implementation, resulting in compliance extensions and the corresponding legal uncertainty. The actual operationalization of the EU AI Act will require a significant expansion in the availability, capacity, and capability of notified bodies. Even if the current EU MDR notified bodies are deemed competent to carry out the performance assessment for medical AI systems (the best-case scenario), there will need to be a significant increase in capacity because all AI/ML-enabled medical devices are considered high-risk AI systems, which require a notified body. Currently, manufacturers of low-risk devices (i.e., Class I) are able to ‘self-certify’ the conformity assessment without or with limited involvement of a notified body. Yet, even Class I (low-risk) medical devices will require a notified body to perform the EU AI Act conformity assessment if they incorporate AI as part of their product. Similarly, the Commission still has to establish the aforementioned ‘simplified technical documentation’ for SMEs and the AI Office facilitate the creation of AI codes of practice. In sum, its ultimate success will largely depend on multiple stakeholders.

Third, the recent developments in large multimodal models and their potential applications in health care may also bring regulatory challenges in the context of medical devices^19^. One potential challenge stems from the “intended use” issue, as some of these models may have multiple uses that may or may not be clearly determined *ex-ante*. With the EU MDR’s applicability based on intended use, the interplay of EU MDR and the EU AI Act might become difficult to navigate. This is especially the case in situations where regulated medical devices are combined with general-purpose AI models developed by a third party. For example, a medical AI device that uses classical ML to analyze medical images to diagnose a medical condition could be *augmented* with a separate general AI, such as a large language model (LLM) in order to enhance the “reasoning” of the medical AI at the front end, as well as to extend its output capabilities (e.g., deliver the diagnosis results in natural language)^20^. The LLM started as a general AI (i.e., an AI with a general intended use), but it is now an AI subsystem within an overall medical system or product whose intended use is a medical diagnosis.

Fourth, the dynamic pace of AI innovation might suffer from a static regulatory approach. Such innovation underscores the necessity of flexible, agile and adaptive regulatory frameworks, capable of accommodating new AI advances, technologies, methodologies, and challenges that have yet to emerge. As an example, the initial version of the EU AI Act did not even contemplate the possibility of generative AI models. Luckily, the protracted negotiations for its approval created the opportunity to include generative AI just before its adoption. The recent introduction of large language models (LLMs) underscores the importance of flexible regulatory frameworks capable of accommodating new AI developments and remaining aligned with technological advancements, societal expectations, and ethical considerations^21^. In fact, the need for last-minute changes in 2023 to the EU Act to account for advances in generative AI—which were entirely uncontemplated in previous versions—helps illustrate how difficult it is for legislation to adapt to rapid technological changes.

All the above concerns are emblematic of the challenges that emerge when horizontal regulation of fast-changing enabling technologies –such as AI– is imposed on sectors with existing regulatory regimes such as regulated digital medical products. For this reason, it will be important that these regulatory interoperability challenges are monitored and addressed in a timely manner before they cause chilling effects on innovation. These underlying concerns have resulted in other jurisdictions, such as the UK, taking a different approach to AI regulation.

The UK has also been playing a leadership role in AI, including hosting the first global AI Safety Summit in November 2023, which brought together the leading AI nations, world leaders, researchers, technology companies, and civil society groups resulting in 28 jurisdictions -including the EU and US- agreeing to The Bletchley Declaration on AI Safety^22^. The UK’s approach to AI regulation, as it diverges from the EU post-Brexit, emphasizes a flexible, pro-innovation framework that allows for sector-specific adaptations by existing regulators. Unlike the EU AI Act’s broad and prescriptive regulations, the UK adopts a principles-based approach focusing on safety and security, transparency, fairness, accountability and governance, and contestability^23^. This approach is underpinned by non-statutory guidance and a three-phased approach to issuing guidelines whereby the various regulators are encouraged to ‘promote innovation and competition’ by developing ‘tools and guidance that promote knowledge and understanding […] in the context of their remit’ by establishing ‘published policy material, in respect of AI, that is consistent with their respective regulatory objectives, setting out clearly and concisely the outcomes regulators expect, so that regulated firms can meet these expectations through their actions.’ Such a regulatory environment in the UK contrasts with the EU’s comprehensive legislative approach but aims equally to manage the multifaceted challenges and opportunities presented by AI technologies. This divergence highlights significant developments in the UK that could influence future AI regulation and its interaction with EU laws. Contrary to the EU, the UK government is taking a ‘deliberately agile and iterative approach, recognising the speed at which these technologies are evolving’ aimed at building the evidence base to learn from experience and continuously adapt to develop a regulatory regime that fosters innovation while ensuring regulatory coherence and addressing emerging AI risks. In fact, according to the 2023 UK Policy Paper “A pro-innovation approach to AI regulation,”^24^ the stated rationale for this pragmatic approach is largely driven by trying to manage the potential risk of hindering AI innovation: “New rigid and onerous legislative requirements on businesses could hold back AI innovation and reduce our ability to respond quickly and in a proportionate way to future technological advances. Instead, the principles will be issued on a non-statutory basis and implemented by existing regulators. This approach makes use of regulators’ domain-specific expertise to tailor the implementation of the principles to the specific context in which AI is used.” For instance, the UK Medicines and Healthcare products Regulatory Agency has issued guidance for ‘AI as a medical device’^25^.

## Conclusions

The EU AI Act applies a comprehensive risk-based approach to regulating digital medical products. By aligning and interacting with existing regulations like the EU MDR/IVDR and GDPR, the Act impacts AI across healthcare, focusing on patient safety, AI system efficacy, data governance, and ethical use. This regulatory landscape is poised to shape the future of AI in healthcare with the goal of fostering innovation while ensuring public trust and compliance. Will this overarching goal be achieved? Given the EU AI Act’s potential impact on AI developments across sectors and internationally, we recommend continuous monitoring and further research. Questions to explore include the EU AI Act’s impact on innovation and SMEs, and the effectiveness of its ‘measures to support innovation’ such as AI regulatory sandboxes. Ensuring the Act is fit for purpose will likely require proactive and evidence-based scholarship, as well as the incorporation of such feedback for its continuous improvement to ensure it achieves its intended purpose.
